# Perspectives for biocatalytic lignin utilization: cleaving 4-*O*-5 and C_α_–C_β_ bonds in dimeric lignin model compounds catalyzed by a promiscuous activity of tyrosinase

**DOI:** 10.1186/s13068-017-0900-3

**Published:** 2017-09-11

**Authors:** Kyoungseon Min, Taewoo Yum, Jiye Kim, Han Min Woo, Yunje Kim, Byoung-In Sang, Young Je Yoo, Yong Hwan Kim, Youngsoon Um

**Affiliations:** 1 Clean Energy Research Center, Korea Institute of Science and Technology (KIST), Seoul, 02792 Republic of Korea; 2 School of Energy and Chemical Engineering, Ulsan National Institute of Science and Technology (UNIST), Ulsan, 44919 Republic of Korea; 3 Department of Chemical Engineering, Hanyang University, Seoul, 04763 Republic of Korea; 4 School of Chemical and Biological Engineering, Seoul National University, Seoul, 08826 Republic of Korea; 5 Present Address: Gwangju Bioenergy Research Center, Korea Institute of Energy Research (KIER), Daejeon, 34129 Republic of Korea; 6 Present Address: Department of Food Sciencen and Biotechnology, Sungkyunkwan University, Suwon, 16419 Republic of Korea

**Keywords:** Sustainable lignin utilization, Tyrosinase, Promiscuous activity, 4-Phenoxyphenol, Guaiacyl glycerol-β-guaiacyl ether (GGE)

## Abstract

**Background:**

In the biorefinery utilizing lignocellulosic biomasses, lignin decomposition to value-added phenolic derivatives is a key issue, and recently biocatalytic delignification is emerging owing to its superior selectivity, low energy consumption, and unparalleled sustainability. However, besides heme-containing peroxidases and laccases, information about lignolytic biocatalysts is still limited till date.

**Results:**

Herein, we report a promiscuous activity of tyrosinase which is closely associated with delignification requiring high redox potentials (>1.4 V vs. normal hydrogen electrode [NHE]). The promiscuous activity of tyrosinase not only oxidizes veratryl alcohol, a commonly used nonphenolic substrate for assaying ligninolytic activity, to veratraldehyde but also cleaves the 4-*O*-5 and C_α_–C_β_ bonds in 4-phenoxyphenol and guaiacyl glycerol-β-guaiacyl ether (GGE) that are dimeric lignin model compounds. Cyclic voltammograms additionally verified that the promiscuous activity oxidizes lignin-related high redox potential substrates.

**Conclusion:**

These results might be applicable for extending the versatility of tyrosinase toward biocatalytic delignification as well as suggesting a new perspective for sustainable lignin utilization. Furthermore, the results provide insight for exploring the previously unknown promiscuous activities of biocatalysts much more diverse than ever thought before, thereby innovatively expanding the applicable area of biocatalysis.

**Electronic supplementary material:**

The online version of this article (doi:10.1186/s13068-017-0900-3) contains supplementary material, which is available to authorized users.

## Background

Biocatalysis is a useful and environmentally friendly approach in the fields of organic chemistry, pharmaceuticals, and biorefinery [1, 2]. To widen the enzymatic functionality of hoped-for reactions, some researchers have aimed to discover an overlooked inherent catalytic potential offered by nature known as catalytic promiscuity [3, 4]. Catalytic promiscuity is the ability of an enzyme to catalyze alternate reactions distinctly different from its primary catalysis [5] and a key factor in the evolution of new enzymatic functions from few ancestral generalist enzymes [3, 6]. Thus, the catalytic promiscuity of enzymes has been shown to be capable of catalyzing the hoped-for reactions through novel biocatalytic routes, thereby expanding the versatility of those enzymes [7].

Depletion of fossil fuels and climate change have accelerated research interest and effort on developing biorefinery using microbial fermentation which utilizes sugars obtained from lignocellulosic biomasses. Fermentable sugars are usually obtained from cellulosic and hemicellulosic components in biomasses by saccharification, whereas lignin is just pretreated for efficient saccharification. Thus, the strategy for utilizing lignin as sustainable feedstock for producing value-added phenolic derivatives is not still enough yet [8]. Even though various chemical pretreatments have been attempted in biorefinery, recently ligninolytic biocatalysts for pretreatment are emerging due to their superior selectivity, lower energy consumption, and unparalleled sustainability [9, 10]. In nature, heme-containing peroxidases such as lignin peroxidase (LiP, E.C. 1.11.1.14), manganese peroxidase (MnP, E.C. 1.11.1.13), versatile peroxidase (VP, E.C. 1.11.1.16), and dye-decolorizing peroxidase (DyP, E.C. 1.11.1.19) are known to be involved in delignification by catalyzing two-electron oxidation requiring a high redox potential of over +1.4 V (vs. normal hydrogen electrode [NHE]) with hydrogen peroxide as an electron acceptor [11, 12]. However, hydrogen peroxide, inevitably required for the catalysis of heme-containing peroxidases, often leads to critical inactivation by attacking and destroying the heme in the active site [13]. Laccase (E.C. 1.10.3.2) catalyzing low- or mid-redox potential compounds (usually < 1.0 V vs. NHE) is sometimes categorized as a ligninolytic biocatalyst; natural (e.g., vanillin) and artificial mediators (e.g., 2,2′-azinobis-(3-ethylbenzthiazoline-6-sulfonate) [ABTS] and 1-hydroxybenzotriazole [HBT]) often enhance the laccase activity for delignification [14]. Besides heme-containing peroxidases and laccase, information on other ligninolytic biocatalysts is very limited to date.

In order to broaden the enzymatic diversity of pretreating lignocellulosic biomasses and to increase the information about ligninolytic biocatalysts, we focused on tyrosinase (E.C. 1.14.18.1) which shows two catalytic activities for various phenolic compounds using O_2_ as the electron acceptor: *o*-hydroxylation of monophenol to diphenol by a cresolase activity and oxidation of diphenol to quinone by a catecholase activity [15]. In addition, we paid special attention to the fact that lignin is a highly branched phenolic-based natural polymer and thus evaluated whether tyrosinase possesses a promiscuous activity associated with delignification. In this study, it was verified that (i) tyrosinase has a promiscuous activity for oxidizing veratryl alcohol which is the most commonly used nonphenolic substrate for assaying ligninolytic activity; (ii) cyclic voltammogram additionally shows the promiscuous activity for oxidizing lignin-related high redox potential substrate; and (iii) the promiscuous activity cleaves the 4-*O*-5 and C_α_–C_β_ bonds in phenolic lignin model dimers independently of cation mediators (e.g., ABTS).

## Results and discussion

### Tyrosinase has a promiscuous activity for oxidizing a lignin-related nonphenolic substrate

**Fig. 1:**
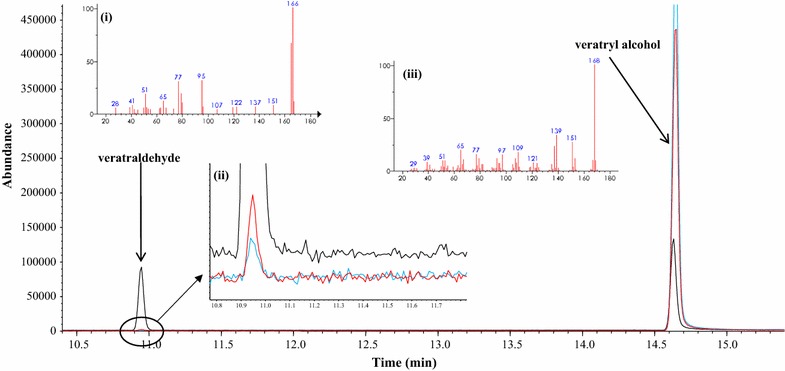
The catalytic promiscuity in tyrosinase oxidizes veratryl alcohol to veratraldehyde. Gas chromatographic (GC) profile and mass spectra of the catalytic product using veratryl alcohol as the substrate in the absence (control, blue line) and presence of 100 unit tyrosinase (reactant, red line). The analyte was extracted with ethyl acetate. The chromatographic peaks were identified by the retention time and mass spectra library (NIST02). Authentic standard chemicals were used to double check the identified substrates and products (standards, black line). Inset: (i) mass spectrum and (ii) magnification of the peak at 10.95 min, and (iii) mass spectrum of the peak at 14.63 min

Veratryl alcohol is the most widely used nonphenolic substrate for assaying ligninolytic activity [16–20], because the redox potential for oxidizing veratryl alcohol to veratraldehyde (>1.4 V vs. NHE) is similar to that for cleaving the β-*O*-4 linkage in lignin [21]. Hence, the promiscuous activity of tyrosinase for delignification was initially examined with veratryl alcohol that is a nonphenolic and thus not a primary substrate for the cresolase and catecholase activities of tyrosinase. The catalytic product was analyzed with GC–MS. As a result, tyrosinase catalyzed the conversion of veratryl alcohol to veratraldehyde, and then the latter was identified by GC–MS with a retention time of 10.95 min (red line in Fig. 1) and a mass spectrum of authentic veratraldehyde (Fig. 1; Additional file 1: Table S1). Because the detection of veratryl alcohol by GC–MS was more sensitive than that of veratraldehyde, the consumed veratryl alcohol was quantified for calculating the degree of conversion and the specific activity, resulting in about 6.2% and 52.5 mU mg^−1^, respectively. Given that tyrosinase oxidized veratryl alcohol to veratraldehyde, tyrosinase was found to have a promiscuous activity for oxidizing a nonphenolic substrate with a high redox potential which is associated with a ligninolytic activity. Table 1 summarizes the kinetic parameters of the promiscuous activity of tyrosinase and LiP a well-known ligninolytic biocatalyst. Tyrosinase oxidized veratryl alcohol with a *k*_cat_ of 0.12 s^−1^ and a *K*_m_ of 0.31 mM, whereas the negative control (no tyrosinase) did not show any absorbance change. LiP was found to have a *k*_cat_ of 13.72 s^−1^ and a *K*_m_ of 3.54 mM. Even though tyrosinase had a tenfold lower catalytic efficiency (*k*_cat_/*K*_m_) than that of LiP due to a lower turnover number (*k*_cat_) and a higher binding affinity (*K*_m_), the promiscuous activity seems to provide an opportunity for exploring a previously unknown versatility of tyrosinase as a ligninolytic biocatalyst [22].

**Table 1:** Kinetic parameters of tyrosinase and lignin peroxidase (LiP) using veratryl alcohol as the substrate

Biocatalyst	*k*_cat_ (s^−1^)	*K*_m_ (mM)	*k*_cat_/*K*_m_ (s^−1^mM^−1^)	Activity (U mg^−1^)
Lignin peroxidase (LiP)	13.72	3.54	3.88	2.06
Tyrosinase	0.12	0.31	0.39	0.058

### Cyclic voltammetry verifies that tyrosinase has a catalytic promiscuity for oxidizing a nonphenolic lignin-related substrate with a high redox potential

**Fig. 2:**
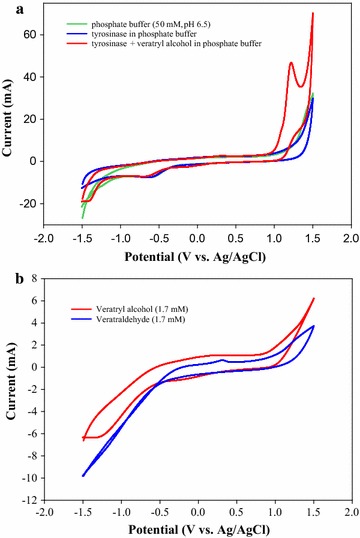
The cyclic voltammetry. **a** The cyclic voltammograms of phosphate buffer (50 mM, pH 6.5) and tyrosinase with and without veratryl alcohol in phosphate buffer (50 mM, pH 6.5). Only in tyrosinase in the coexistence of veratryl alcohol, the oxidation peak was shown at +1.22 V vs. Ag/AgCl, which might be corresponding to +1.42 V vs. NHE. **b** The cyclic voltammogram of veratryl alcohol (1.7 mM) and veratraldehyde (1.7 mM) in phosphate buffer (pH 6.5). NO oxidation and reduction peaks were observed in each of veratryl alcohol and veratraldehyde. In all the cyclic voltammetries, glassy carbon, coiled Pt wire, and Ag/AgCl electrode were used as the working, counter, and reference electrode, respectively. Cyclic voltammetry was carried out using potentiostat/galvanostat controlled by commercial WMPG software. The scan rate was 50 mVs^−1^

To further study the redox potential of tyrosinase during veratryl alcohol oxidation, cyclic voltammetry was performed. Figure 2a represents that tyrosinase itself did not show any oxidation peaks, but tyrosinase with veratryl alcohol as the substrate exhibited a definite oxidation peak at +1.22 V (vs. Ag/AgCl). As shown in Fig. 2b, no oxidation peaks were observed in the cyclic voltammograms of veratryl alcohol and veratraldehyde alone. Given that tyrosinase, veratryl alcohol, and veratraldehyde were not oxidized under the scan range (−1.5 to +1.5 V), the oxidation peak appearing at +1.22 V (vs. Ag/AgCl) in the mixture of tyrosinase and veratryl alcohol might be the result of tyrosinase-driven veratryl alcohol oxidation. When tyrosinase catalyzed veratryl alcohol oxidation, deprotonation of the veratryl alcohol was detected at the anode, and subsequently a definite oxidation peak was observed. Additionally, the oxidation potential (+1.22 V vs. Ag/AgCl corresponding to +1.43 V vs. NHE) was consistent to the oxidation potential of LiP for veratryl alcohol oxidation (>1.4 V vs. NHE) [21]. Consequently, the cyclic voltammogram shown in Fig. 2a provides evidence confirming that tyrosinase has the catalytic promiscuity for oxidizing nonphenolic lignin-related substrate with the high redox potential (i.e., veratryl alcohol).

### The promiscuous activity of tyrosinase cleaves the 4-*O*-5 and C_α_–C_β_ bond in dimeric lignin model compounds

**Fig. 3:**
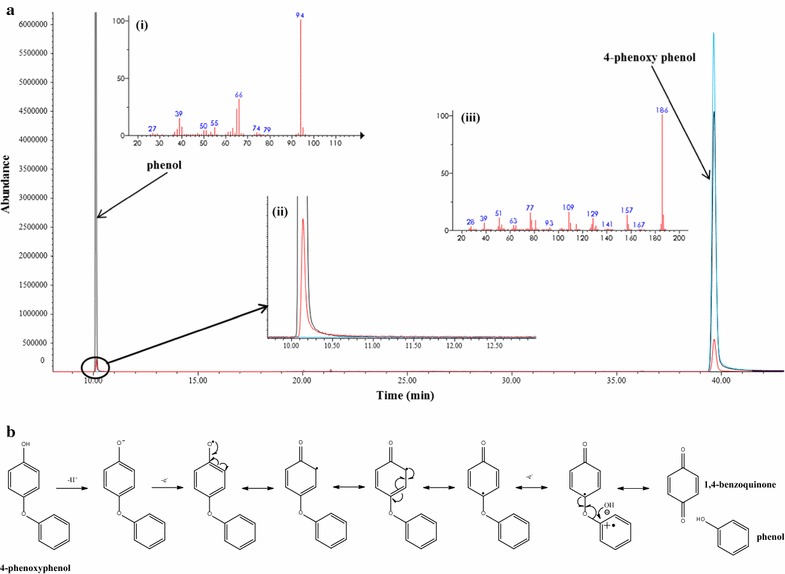
Cleavage of 4-*O*-5 bond in 4-phenoxyphenol by the catalytic promiscuity of tyrosinase. **a** GC profile and mass spectra (MS) of the catalytic product using 4-phenoxyphenol in the absence (control, blue line) and presence of 100 unit tyrosinase (reactant, red line). The analyte was extracted with ethyl acetate. The chromatographic peaks were identified by the retention time and mass spectra library (NIST02). The authentic standard chemicals were used to confirm the identified (standard, black line). Inset: (i) mass spectrum and (ii) magnification of the peak at 10.15 min, and (iii) mass spectrum of the peak at 39.7 min. **b** Reaction scheme illustrates the catalytic promiscuity cleaves the 4-*O*-5 bond in 4-phenoxyphenol. The catalytic product phenol is a primary substrate of tyrosinase (cresolase activity)

In order to verify whether the promiscuous activity of tyrosinase is able to cleave linkages in lignin, dimeric lignin model compounds were tested as substrates. 4-Phenoxyphenol [23] and guaiacyl glycerol-β-guaiacyl ether (GGE) [24] were used as the dimeric lignin model compounds to represent the 4-*O*-5 and β-*O*-4 linkages in lignin, respectively. When using 4-phenoxyphenol as the substrate, phenol was produced as the catalytic product (Fig. 3a; Additional file 1: Table S1). In addition, the clear 4-phenoxyphenol solution turned brown, and then, a dark brown precipitate was observed in a time-dependent manner. This phenomenon can be explained by quinone polymerization. The promiscuous activity of tyrosinase appeared to oxidatively cleave the 4-*O*-5 bond in 4-phenoxyphenol, thereby producing phenol and possibly 1,4-benzoquinone as shown in Fig. 3b. Phenol is a well-known primary substrate of tyrosinase and thus was sequentially converted to catechol and 1,2-benzoquinone by the cresolase and catecholase activities, respectively. Then, the 1,2-benzoquinone appeared to be polymerized through spontaneous oxidation, resulting in a dark brown precipitate [25]. In addition, we aimed to validate the 1,4-benzoquinone in the reaction sample. However, the authentic 1,4-benzoquinone was found to be unstable in the reaction conditions (pH 6.5 at 30 °C) and a small amount of brown precipitate was observed, which made it difficult to validate 1,4-benzoquinone in the reaction sample. To determine the degree of conversion and the specific activity, the consumed 4-phenoxyphenol was quantified by GC–MS and 3.04 mg L^−1^ of 4-phenoxyphenol was detected after a 24-h reaction, thereby indicating that the conversion rate and the specific activity for 4-phenoxyphenol were about 96.7% and 0.77 U mg^−1^, respectively.

**Fig. 4:**
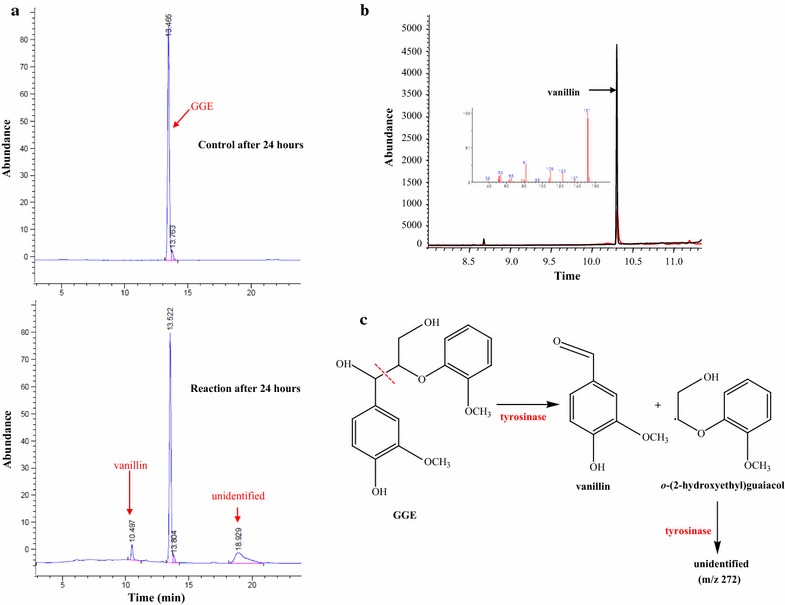
Decomposition of guaiacyl glycerol-β-guaiacyl ether (GGE) by the catalytic promiscuity of tyrosinase. **a** HPLC profile of GGE and vanillin in the control without tyrosinase and in the reaction sample which includes tyrosinase. The chromatographic peaks were identified by the retention time and authentic standard chemicals. **b** GC–MS of the catalytic product when using GGE as the substrate in the presence of 500 unit tyrosinase (red line). Black line represents authentic vanillin standard. Inset: mass spectrum of the peak at 10.31 min **c** Reaction scheme illustrates that the catalytic promiscuity cleaves the C_α_–C_β_ bond in GGE

Furthermore, GGE, a dimeric lignin model compound that includes a β-*O*-4 bond, which is the most prevalent linkage in lignin (>50%) [26], might be decomposed by the promiscuous activity of tyrosinase. As shown in Fig. 4a, one of the catalytic products was detected at 10.497 min in high performance liquid chromatography (HPLC) profile which was the same retention time as that of authentic vanillin. The estimated catalytic product vanillin was additionally verified by GC–MS (Fig. 4b; Additional file 1: Table S1). Furthermore, we quantified the produced vanillin; after a 24-h reaction, 12.28 mg L^−1^ of vanillin were detected, thereby representing a conversion, specific activity, and vanillin production rate of 16.1%, 1.3 mU mg^−1^, 0.078 μmol h^−1^ mg^−1^, respectively. Vanillin is a useful compound which can be obtained from lignocellulose, but the information about enzymatic conversion of lignocellulose to vanillin has been limited. Instead, there are several reports about microbial vanillin production from lignocellulose: Kumar et al. utilized a natural bacterial consortium for valorizing lignocellulosic biomass and produced vanillin with the conversion of 0.36% [27]. In addition, vanillin dehydrogenase-deleted *Rhodococcus jostii* RHA1 has been reported to convert 0.38% of wheat straw lignocellulose to vanillin [28].

Because the substrate, GGE, was not detected under the GC–MS conditions used for the vanillin analysis (using a HP-Innowax column), derivatization with tetramethylsilane (TMS) was carried out to analyze GGE. As a result, GGE-TMS was detected at a retention time of 26.1 in a GC–MS equipped with a HP-Ultra2 column (Additional file 1: Fig. S1, Table S1). In the HPLC profile (Fig. 4a), one unidentified peak at 18.929 min was also observed in the reaction sample. GC–MS analysis indicated that the estimated *m/z* value of the unidentified product was of 272 (Additional file 1: Table S1). It is not clear how the unidentified product was formed. A possible mechanism would be a further conversion of catalytic product (other than vanillin) to an unidentified product by tyrosinase. In accordance with Ahmad et al., bacterial DyP, one of the ligninolytic heme-containing peroxidases, catalyzes GGE to vanillin and guaiacol [12]. In addition, Chen et al. validated that DyP from *Thermomonospora curvata* would have a potential for lignin degradation and proposed the catalytic mechanism for decomposing GGE to guaiacol oligomer [29]. Besides DyP, ligninolytic heme-containing peroxidases have been known to easily convert guaiacol to guaiacol-oligomer [12, 19, 24]. As with DyP from *Rhodococcus jostii* RHA1 [12], the catalytic promiscuity of tyrosinase seemed to cleave the C_α_–C_β_ bond in GGE, yielding vanillin (Fig. 4c) and possibly an unstable *o*-(2-hydroxyethyl)guaiacol radical. The unstable *o*-(2-hydroxyethyl)guaiacol radical might be further catalyzed to guaiacol and 2-hydroxyacetaldehyde by the tyrosinase, and then guaiacol might be polymerized to an unidentified product with a *m/z* of 272 as shown in Fig. 4c: given that (i) the reduction of compound II to resting state in DyP initiates the conversion of *o*-(2-hydroxyethyl)guaiacol to guaiacol [12] and (ii) the redox potential of compound II/resting state in peroxidases is estimated ranging from +0.93 to +1.26 V [30], the promiscuous activity of tyrosinase oxidizing a high redox potential substrate veratryl alcohol (+1.22 V) might convert *o*-(2-hydroxyethyl)guaiacol to guaiacol. Additionally, Garcia-Molina et al. demonstrated the catalytic activity of tyrosinase on guaiacol [31]. Therefore, the promiscuous activity of tyrosinase seemed to directly cleave the C_α_–C_β_ bond according to vanillin formation and possibly guide the cleavage of the β-*O*-4 bond in GGE. However, further identification on the degradation products of GGE by tyrosinase is required to elucidate the degradation mechanism.

## Conclusion

Given that tyrosinase exhibits a broad substrate specificity for various phenolic compounds and that lignin is a highly branched phenolic-based natural polymer, we herein explored the novel promiscuous activity of tyrosinase closely associated with delignification. Tyrosinase not only oxidizes veratryl alcohol, the most widely used nonphenolic substrate for assaying ligninolytic activity, but also cleaves the 4-*O*-5 and C_α_–C_β_ bonds in dimeric lignin model compounds. Consequently, tyrosinase seems to be applicable for biocatalytic pretreatment in biorefinery utilizing lignocellulosic biomass as a sustainable feedstock. Furthermore, the results provide a new perspective for sustainable lignin utilization to produce value-added phenolic derivatives.

Even if higher substrate specificity is a unique feature of a biocatalyst distinguishable from a chemical catalyst, recently reported promiscuous activities have been considered as favorable properties in numerous applications [22]. In accordance with Nam et al., the higher substrate specificity in a biocatalyst is a result of the evolution from promiscuous activities in ancestral generalist biocatalysts [6]. In this study, we, for the first time, identified the valuable catalytic promiscuity of tyrosinase associated with delignification. The results discussed herein could extend the versatility of tyrosinase as a promising ligninolytic biocatalyst under mild operational conditions (neutral pH using O_2_ as the electron acceptor). Given that the higher substrate specificity in a biocatalyst is a result of the evolution from promiscuous activities [6], the results provide insight into further understanding of how the lignocellulose-degrading biocatalytic specificity has evolved from promiscuous ancestral biocatalysts which might be much more diverse than ever thought before.

Tyrosinase has been studied for a long time in various fields including in bioremediation for detoxifying phenolics [32], in pharmaceutics for L-DOPA production [15, 33, 34], and in bioelectronics for detecting phenolics [35]. Nevertheless, the structure-based catalytic mechanism of tyrosinase has not been actively discussed until Matoba et al. reported the X-ray crystal structure of tyrosinase from *Streptomyces castaneoglobisporus* [36]. Based on the recently verified structure of tyrosinase from *Agaricus bisporus* (PDB ID:2Y9W) [37, 38], we will aim to suggest a catalytic mechanism for the promiscuous activity in the near future.

## Methods

### Enzymes and chemicals

Tyrosinase from *Agaricus bisporus*, LiP from *Phanerochaete chrysosporium*, veratryl alcohol (96%), veratraldehyde (99%), 4-phenoxy phenol (99%), benzyl phenyl ether (98%), dimethyl sulfoxide (DMSO, 99%), and benzyl benzoate (99%) were purchased from Sigma-Aldrich (St. Louis, USA). GGE (97%) and trimethylchlorosilane (TMCS, 1%) in *N,O*-bis(trimethylsilyl) trifluoroacetamide (BSTFA) [BSTFA + TMCS] were purchased from Tokyo Chemical Industry Co., Ltd (Tokyo, Japan) and Supelco analytical (Bellefonte, USA), respectively. To remove trace impurities such as metal salts, tyrosinase was solubilized in phosphate buffer (50 mM, pH 6.5) and then purified with an ultrafiltration device with a cutoff of 10 kDa (Merck Millipore, MA, USA). All other chemicals were used as the highest grade available without further purification.

### Reaction

The catalytic promiscuity of tyrosinase was tested with the following substrates: 10 mM veratryl alcohol. Due to the low solubility in the aqueous phase, 0.5 mM of 4-phenoxyphenol and GGE were used in phosphate buffer (pH 6.5) and 0.625% DMSO, respectively. The reaction was initiated by adding 100 (for veratryl alcohol and 4-phenoxyphenol) or 500 U (for GGE) of tyrosinase into 1 mL of a substrate solution in 50 mM phosphate buffer (pH 6.5). One U of tyrosinase converts 1 μmol of l-tyrosine to L-DOPA per min at pH 6.5 and 25 °C. All reactions were conducted at 30 °C for 24 h.

### GC–MS analysis

After a 24-h reaction, the catalytic products from veratryl alcohol, 4-phenoxyphenol, and GGE were identified by GC–MS. The analytes were extracted with ethyl acetate (for veratryl alcohol, veratraldehyde, 4-phenoxyphenol, and phenol) or methyl chloride (for GGE and vanillin), and then, GC–MS analysis was performed with a 6890N GC interfaced with a 5975 MS (Agilent Technologies, Santa Clara, USA) under the following conditions: a fused polyethylene glycol HP-INNOWax capillary (30 m × 0.25 mm i.d., 0.25 μm, Agilent Technologies, Santa Clara, USA) and He (99.9999%) were used as the column and carrier gas at a constant flow rate (1.2 mL min^−1^), respectively. To analyze GGE, TMS derivatization was performed with BSTFA + TMCS, and a HP-Ultra2 column was used. Mass spectrometry was conducted in the electron impact mode (70 eV), and the ion source temperature was 230 °C. The temperature program of the oven is summarized in Additional file 1: Table S2. Chromatographic peaks were identified by the retention time and the mass spectra library (NIST02). Authentic standard chemicals were used to confirm the identification.

### Cyclic voltammetry

A one-compartment electrochemical cell was constructed with a 3-electrode system for cyclic voltammetry. Glassy carbon, coiled Pt wire, and Ag/AgCl were used as the working, counter, and reference electrodes, respectively. Cyclic voltammetry was carried out using a potentiostat/galvanostat (WMPG1000K8, WonA Tech., Seoul, Korea) controlled by commercial WMPG software. In all cyclic voltammetries, the scan rate and the scan range were 50 mV s^−1^ and −1.5 to +1.5 V, respectively.

### Kinetics

To determine the kinetic parameters, various concentrations of veratryl alcohol (0.1, 0.25, 0.5, 1.0, 2.0, and 5.0 mM) were used, and the initial increase of absorbance was monitored at 310 nm (ε_veratraldehyde,310 nm_ = 9.3 mM^−1^ cm^−1^) with a UV-spectrophotometer (Shimazhu, UV-1240, Kyoto, Japan) at 30 °C [19]. To calculate the kinetic parameters shown in Table 1, the kinetic data were fitted into the Michaelis–Menten equation.

### HPLC analysis

GGE and vanillin were analyzed by HPLC (Agilent 1200 HPLC system, CO, USA). The HPLC procedure was performed by injecting fractions using a reverse-phase Eclipse XDB-C18 column (4.6 × 150 mm, 5 μm, Agilent). Gradient separation was performed from distilled water (solvent A) to methanol (solvent B) using the following conditions: flow rate 1.0 mL min^−1^, column temperature 25 °C, time 0 min-5% B, time 5 min-25% B, time 10 min-40% B, time 30 min-50% B, time 35 min-100% B. Using a UV detector at 280 nm, authentic GGE and vanillin were detected at 13.522 and 10.497 min, respectively.

## Additional file

**Additional file 1.** Additional figure and tables.
