# Supplementary material for: Perspectives for biocatalytic lignin utilization: cleaving 4-O-5 and Cα–Cβ bonds in dimeric lignin model compounds catalyzed by a promiscuous activity of tyrosinase
Source: Biotechnol Biofuels. 2017 Sep 11;10:212. doi: 10.1186/s13068-017-0900-3 (PMC5594458; doi:10.1186/s13068-017-0900-3)
Supplement: Supplementary file 1 — Additional file 1. Additional figure and tables. [file 13068_2017_900_MOESM1_ESM.docx]

**Additional Information**

**Perspective for biocatalytic lignin utilization: cleaving 4-O-5 and C_α_-C_β_ bonds in dimeric lignin model compounds catalyzed by a promiscuous activity of tyrosinase**

Kyoungseon Min^1,2^, Taewoo Yum^1^, Jiye Kim^1^, Han Min Woo^1^, Yunje Kim^1^, Byoung-In Sang^3^, Young Je Yoo^4^, Yong Hwan Kim^2^, and Youngsoon Um^1*^

^1^Clean Energy Research Center, Korea Institute of Science and Technology (KIST), Seoul 02792, ^2^School of Energy and Chemical Engineering, Ulsan National Institute of Science and Technology (UNIST), Ulsan 44919, ^3^Department of Chemical Engineering, Hanyang University, Seoul 04763, ^4^School of Chemical and Biological Engineering, Seoul National University, Seoul 08826, Korea

*corresponding author, Dr. Youngsoon Um

Phone : +82-2-958-5819, Fax : +82-2-958-6858, E-mail : [yum@kist.re.kr](mailto:yum@kist.re.kr)

(a)


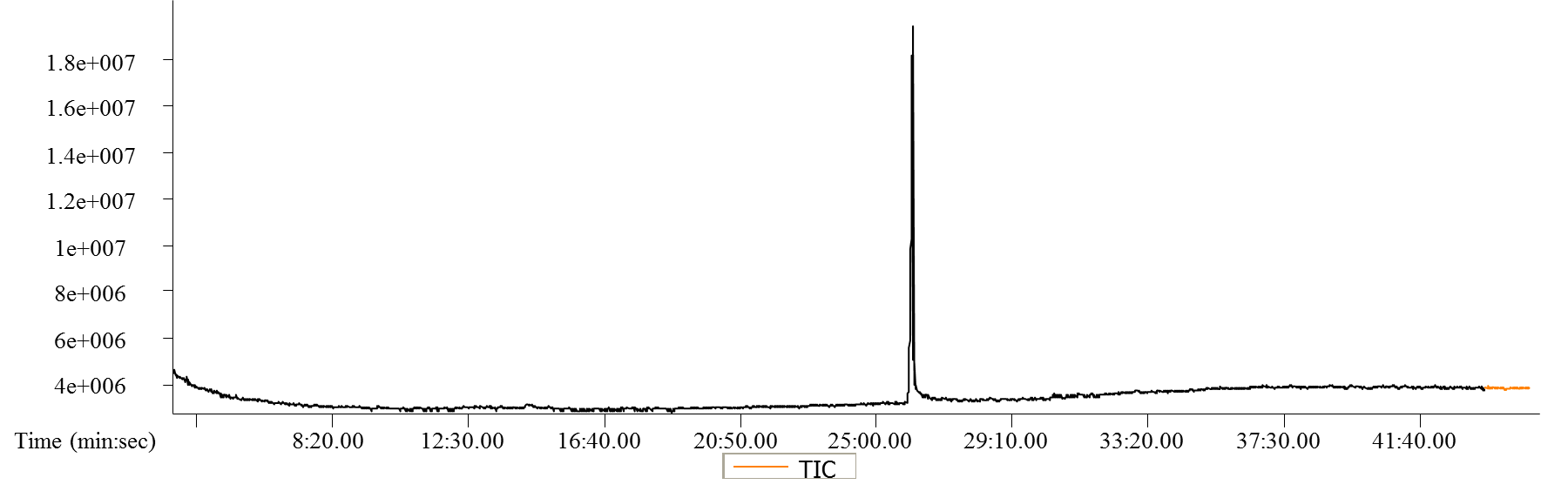


(b)


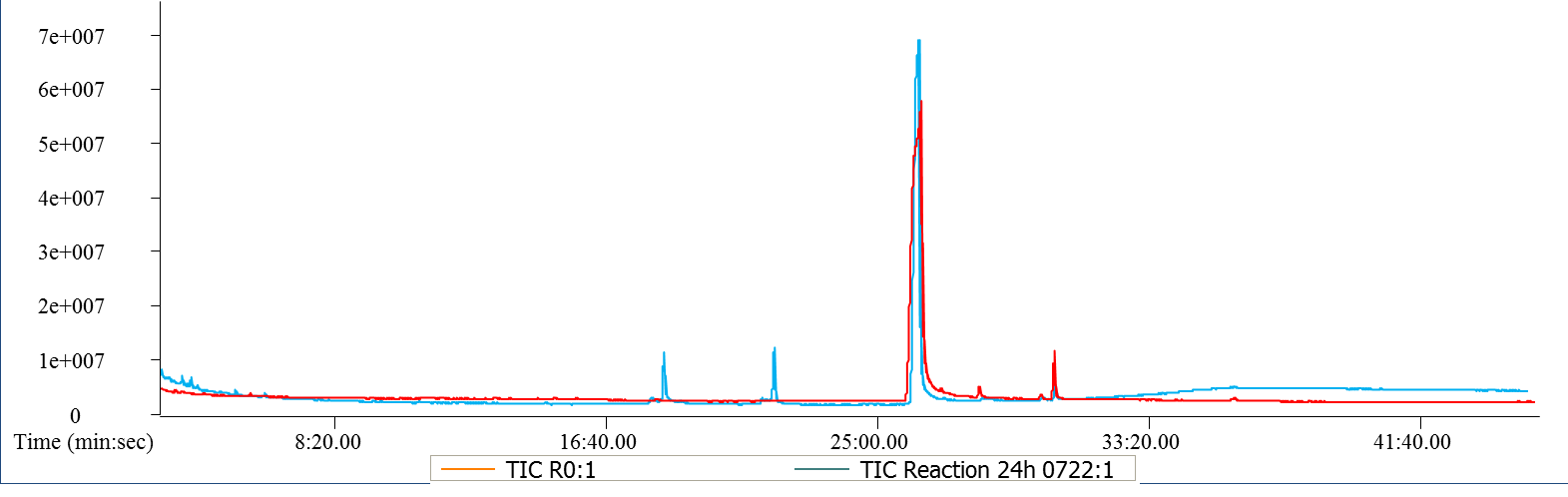


**Fig.S1** **(a)** GC-MS of authentic TMS-derivatized GGE (GGE-TMS) using HP-Ultra2 column. GGE-TMS detected at 26.1 min. **(b)** GC-MS of GGE-TMS in the reaction sample. Blue and red line represents the initial (t=0) and final (t=24 hours) GC profile of reaction sample, respectively

**Table S1**. Mass spectrum comparison of real mass in the reaction samples and standard mass of authentic standard chemicals

|  | **Real mass** | **Standard mass** |
| --- | --- | --- |
| veratryl alcohol |  | 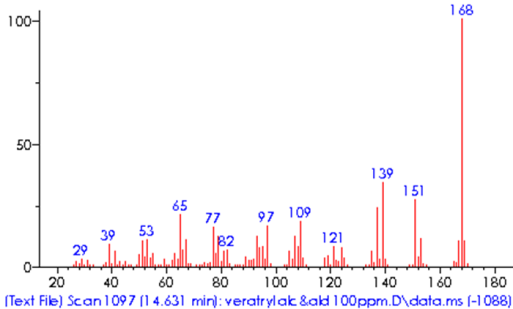 |
| veratryl aldehyde |  | 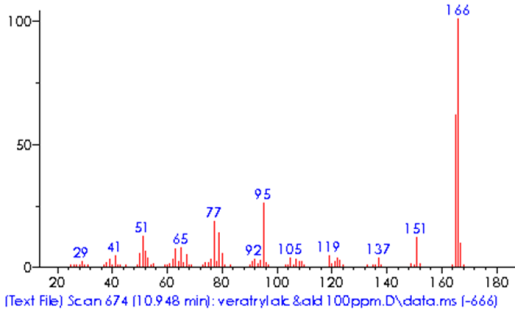 |
| 4-phenoxyphenol | 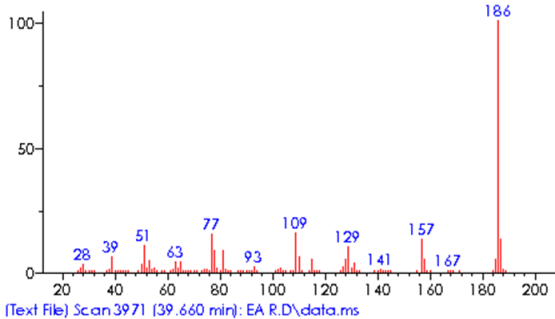 | 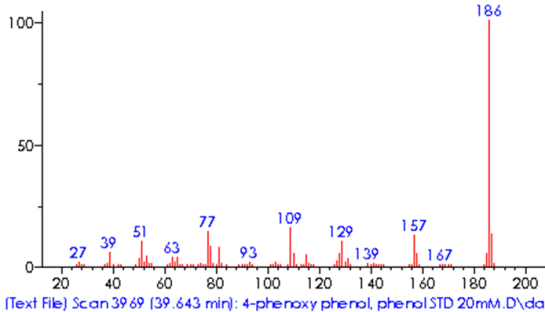 |
| phenol | 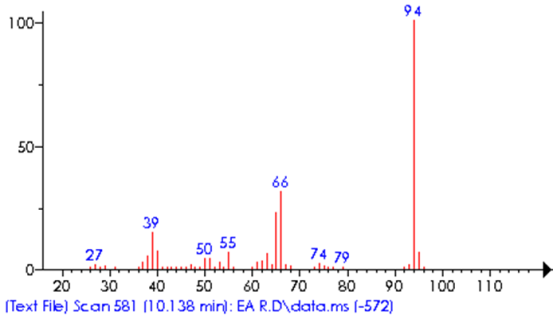 | 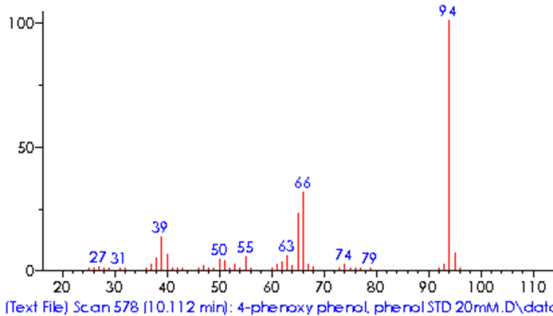 |
| vanillin | 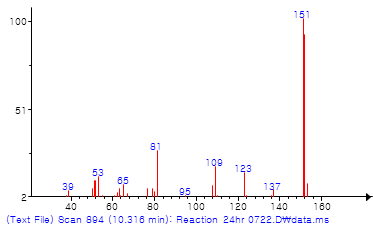 | 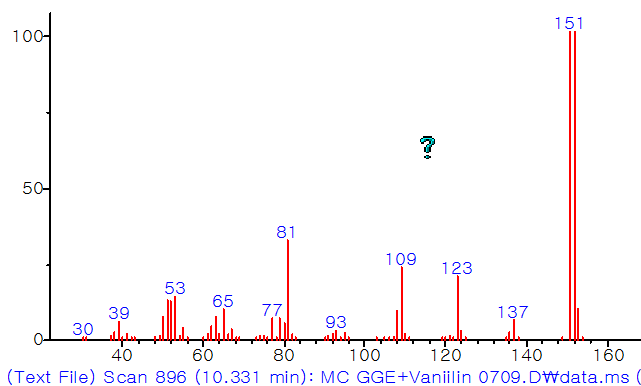 |
| GGE-TMS |  |  |
| Unidentified | 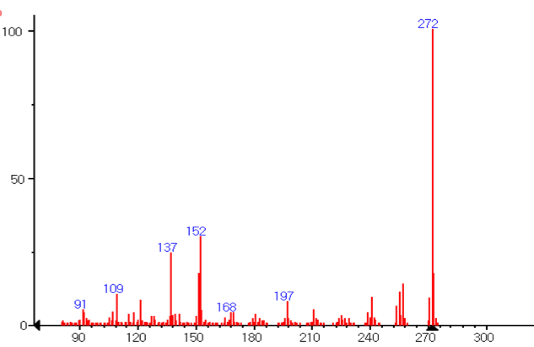 |  |

**Table S2**. Temperature program of oven for GC-MS analysis

| **Analyte** | **Temperature program** | | | |
| --- | --- | --- | --- | --- |
|  | **Initial (^o^C)** | **Final (^o^C)** | **Rate (^o^Cmin^-1^)** | **Holding time (min)** |
| Veratryl alcohol, veratryl aldehyde | 160 | 240 | 5 | 20 |
| Phenol, 4-phenoxyphenol | 120 | 220 | 5 | 30 |
| Vanillin | 50 | 250 | 20 | 6 |
| GGE-TMS | 100 | 270 | 5 | 6 |
